# The Effect of *Babesia divergens* Infection on the Spleen of Mongolian Gerbils

**DOI:** 10.1155/2014/483854

**Published:** 2014-07-17

**Authors:** Mohamed A. Dkhil, Saleh Al-Quraishy, Mohamed S. Al-Khalifa

**Affiliations:** ^1^Department of Zoology, College of Science, King Saud University, P.O. Box 2455, Riyadh 11451, Saudi Arabia; ^2^Department of Zoology and Entomology, Faculty of Science, Helwan University, Cairo 11795, Egypt

## Abstract

Babesiosis is caused by intraerythrocytic protozoan parasites transmitted by ticks and affects a wide range of domestic and wild animals and occasionally humans. The current study aimed to investigate the effect of *B. divergens* infected erythrocytes on spleen histopathology, cell cycle alteration, and the presence of oxidative stress. Mongolian gerbils were challenged with 5 × 10^6^ *Babesia divergens* infected erythrocytes. Parasitemia reached approximately 77% at day 5 postinfection. Infection also induced injury of the spleen. This was evidenced with (i) increases in cellular damage of the spleen, (ii) decrease in antioxidant capacity as indicated by decreased glutathione, catalase, and superoxide dismutase levels, (iii) increased production of malondialdehyde and nitric oxide derived products (nitrite/nitrate), and (iv) increased lactic acid dehydrogenase activity and protein carbonyl content in the spleen. Infection interfered with normal cell cycle of the spleen cells at G_0_/G_1_, S, and G_2_/M phases. On the basis of the above results it can be hypothesized that *B. divergens* infected erythrocytes could alter the spleen histopathology and cause cell cycle alteration and induce oxidative stress in splenic tissue.

## 1. Introduction

Babesiosis is a zoonotic infection in which ticks transmit* Babesia* organisms from a vertebrate reservoir to humans; the infection is incidental in humans [1]. The clinical signs and symptoms of babesiosis are related to the parasitism of red blood cells (RBCs) by* Babesia.* Fever, hemolytic anemia, and hemoglobinuria may result from* Babesia* infection [2].

Members of the genus Babesia are intraerythrocytic protozoan parasites, and many species are of considerable economic importance in the livestock industry. Additionally, some species affect human health [3].* B. divergens* is transmitted by* Ixodes ricinus*, a member of the family of hard ticks (*Ixodidae*). Recently, worldwide interest in* B. divergens* has increased as a result of human cases caused by identical or similar parasites outside areas where bovine babesiosis is endemic [4]. As with malaria, cells of the reticuloendothelial system in the spleen remove damaged RBC fragments from the circulation [5].

Many parasites including protozoa are sensitive to oxidative stress. Sensitivity to oxidative stress has been reported in malaria [6], hepatozoonosis [7], tropical theileriosis [8], and babesiosis [9]. Free radicals and other reactive oxygen species (ROS) have been implicated to play an important role in tissue damage in a variety of pathological processes [6]. Overproduction of ROS in diverse pathological conditions leads to oxidative damage to macromolecules resulting in enhanced lipid peroxidation and DNA strand breaks [10]. To counteract oxidative damage caused by ROS generated during infections, there is generation of multilayered defense system including DNA repairs systems, scavenging substrates, and antioxidant enzyme system [10].

Although some parasites can induce DNA damage, there are no enough data on* B. divergens *genotoxicity. The current study aimed to investigate the effect of* B. divergens* infected erythrocytes on spleen histopathology, cell cycle alteration, and the presence of oxidative stress.

## 2. Materials and Methods

### 2.1. Infection of Gerbils

*Babesia divergens* was kindly provided by Professor Mehlhorn (Heinrich Heine University, Duesseldorf, Germany). This strain has been maintained in our laboratory in Mongolian gerbils (*Meriones unguiculatus*) by intraperitoneal injection of infected blood.

Male* M. unguiculatus* aged from 9 to 11 weeks old were used. They were bred under specific pathogen-free conditions in the animal facilities of King Saud University, Riyadh, Saudi Arabia. They were housed in plastic cages and fed on standard diet and given water* ad libitum*.

To determine the extent of parasitaemia and survival of gerbils, 12 animals were inoculated with 5 × 10^6^ *B. divergens*-parasitized erythrocytes. Parasitaemia was evaluated using Giemsa-stained smears, which were prepared daily using blood taken from the tail veins, and the total number of parasitized erythrocytes per mL was estimated by counting in a Neubauer chamber. To study the spleen injury induced by the parasite in gerbils, it was necessary to allow infections to progress until parasitaemia had reached 60–70%. Eight animals that had been infected with 5 × 10^6^ *B. divergens*-parasitized erythrocytes were anaesthetized with ether and then killed by cervical dislocation on day 5 postinfection (p.i.), along with 8 noninfected control animals. Parasitemia was evaluated in Giemsa-stained blood smears from the tail veins. The experiments were approved by the state authorities and followed Saudi Arabian rules on animal protection (Project number RGP-198).

### 2.2. Histopathology

Small pieces of spleen were fixed in 10% neutral buffered formalin. Specimens were then routinely dehydrated with ethanol and embedded in paraffin, and then 5 *μ*m paraffin sections were cut and stained with hematoxylin and eosin for histological study. A semiquantitative scoring system [11] was used. Spleen was scored for the enlargement of B- and T-lymphocyte areas in red and white pulps (0, absent; 1, slight; 2, moderate; and 3, pronounced) and for the increased number of apoptotic cells, macrophages, necrotic cells, and presence of pigments (0, absent and 1, present). Scoring of each tissue sample represented the mean score of five different high microscopic power fields. Stained tissue sections were imaged using light microscope (Olympus, Japan) provided with digital camera with a high resolution.

### 2.3. Biochemical Analysis

Spleen was weighed and homogenized immediately to give 50% (w/v) homogenate in ice-cold medium containing 50 mM Tris-HCl and 300 mM sucrose [12]. The homogenate was centrifuged at 500 g for 10 min at 4°C. The supernatant (10%) was used for the various biochemical determinations.

### 2.4. Glutathione

Glutathione (GSH) was determined chemically in spleen homogenate using Ellman's reagent [13]. The method is based on the reduction of Ellman's reagent (5,5_dithiobis (2-nitrobenzoic acid) with GSH to produce a yellow compound. The chromogen is directly proportional to GSH concentration, and its absorbance was measured at 405 nm.

### 2.5. Activities of Catalase and Superoxide Dismutase

Catalase activity was assayed by the method of Aebi [14]. Catalase reacts with a known quantity of H_2_O_2_. The reaction is stopped after exactly one minute with a catalase inhibitor. In the presence of horseradish peroxidase, remaining H_2_O_2_ reacts with 3,5-dichloro-2-hydroxybenzenesulfonic acid and 4-aminophenazone to form a chromophore with a color intensity inversely proportional to the amount of catalase in the original sample, measured at 240 nm. Superoxide dismutase activity in plasma was assayed by the method of Nishikimi et al. [15]. This assay relies on the ability of the enzyme to inhibit the phenazine methosulphate-mediated reduction of nitroblue tetrazolium dye, which was measured at 560 nm.

### 2.6. Malondialdehyde

Lipid peroxidation in spleen homogenate was determined according to the method of Ohkawa et al. [16] by using 1 mL of trichloroacetic acid 10% and 1 mL of thiobarbituric acid 0.67%, followed by heating in a boiling water bath for 30 min. Thiobarbituric acid reactive substances were determined by the absorbance at 535 nm and expressed as malondialdehyde (MDA) equivalents formed.

### 2.7. Nitrite

The assay of nitrite in spleen homogenate was done according to the method of Berkels et al. [17]. In acid medium and in the presence of nitrite the formed nitrous acid diazotises sulphanilamide, which is coupled with N-(1-naphthyl) ethylenediamine. The resulting azo dye has a bright reddish-purple color which was measured at 540 nm.

### 2.8. Lactate Dehydrogenase Cytotoxicity Assay

Lactate dehydrogenase (LDH) assay was performed in the spleen homogenate by using a commercial kit based on the transformation of pyruvate to lactate by LDH, at pH 7.5, in the presence of NADH coenzyme. The transformation of NADH to NAD^+^ is accompanied by a decrease in absorbance (*A*) at 340 nm, which correlates with the LDH activity. The change of absorbance, in the absence or presence of parasite, was recorded over a 0.5 to 4.5 min period, and the relative Δ*A*/min was calculated. The change in absorbance was converted to LDH international units per liter (U/l) by the following calculations: Δ*A*/min (tV × 1,000/EMC × *l*  X  sV), where tV is the total volume, EMC is the NADH extinction micromolar coefficient (6.22 cm^2^ *μ*mol at 340 nm),* l* is the light path length (1 cm), and sV is the sample volume. The final activity was expressed as U/g protein.

### 2.9. Determination of Protein Carbonyl Content

Protein carbonyl content was determined as described by Levine et al. [18], with slight modifications. Spleen homogenate was incubated with 0.5 mL of 10 mM dinitrophenylhydrazine in 2 M HCl, for 1 h at room temperature in the dark with occasional mixing. The protein hydrazone derivatives were precipitated with 0.5 mL of 20% trichloroacetic acid and the precipitates were washed three times with 1 mL ethanol : ethyl acetate (1 : 1). During each washing, the homogenized pellet was vortexed and left in the washing solution for 10 min at room temperature before centrifugation. The final pellet was resuspended in 6 M guanidine HCl and incubated for 15 min at 37°C. The carbonyl content was determined spectrophotometrically at 360 nm, on the basis of molar absorbance coefficient of 22,000 M^−1^ cm^−1^.

### 2.10. Cell Cycle and DNA Damage Analysis by Flow Cytometry

The cell cycle and DNA damage were evaluated with propidium iodide (PI) staining and flow cytometry according to the method previously described by Hishikawa et al. [19]. Propidium iodide is a specific fluorescent dye that stains the double-stranded DNA. In methanol-fixed cells, the PI molecules translocate into the nucleus and bind to the double-stranded DNA. The DNA fluorescence of PI-stained cells was analyzed by excitation at 488 nm and monitored through a 630/22 nm band-pass filter using a FACScan flow cytometer (Becton-Dickinson, Frankton Lakes, NJ). In brief, noninfected and infected spleen tissues were each homogenized and washed with PBS and then centrifuged at 200 ×g for 5 min. Spleens were treated with proteolytic enzymes (trypsin) to digest proteins in the extracellular matrix and chelate calcium responsible for cell-cell adhesion with ethylenediaminetetraacetic acid (EDTA). After 1-2 hrs of enzymatic treatment in EDTA buffer, the tissue can then be teased or gently shaken apart into single living cells. The cell pellets were fixed in PBS-methanol (1 : 2, volume/volume) solution and then maintained at 4°C for at least 1 h. After one wash with PBS, the cell pellets were stained with a fluorescent probe solution containing PBS, 50 *μ*g mL^−1^ PI, and 50 *μ*g mL^−1^ DNase-free RNase A. The suspension was incubated for 30 min at room temperature in the dark and filtered through 200 *μ*m nylon mesh. The DNA fluorescence of PI-stained cells was analyzed by excitation at 488 nm and monitored through a 630/22 nm band-pass filter using a FACScan flow cytometer (Becton-Dickinson, USA). A minimum of 10000 cells was counted per sample, and the DNA histograms were evaluated further using cell Quest software on a PC workstation to calculate the percentage of cells in various phases of the cell cycle.

### 2.11. Statistical Analysis

Statistical analyses were performed by Student's *t*-test using a statistical package program (SPSS version 17.0). *P* values are ≤0.05 considered as significant for all statistical analyses in this study.

## 3. Results

**Figure 1:**
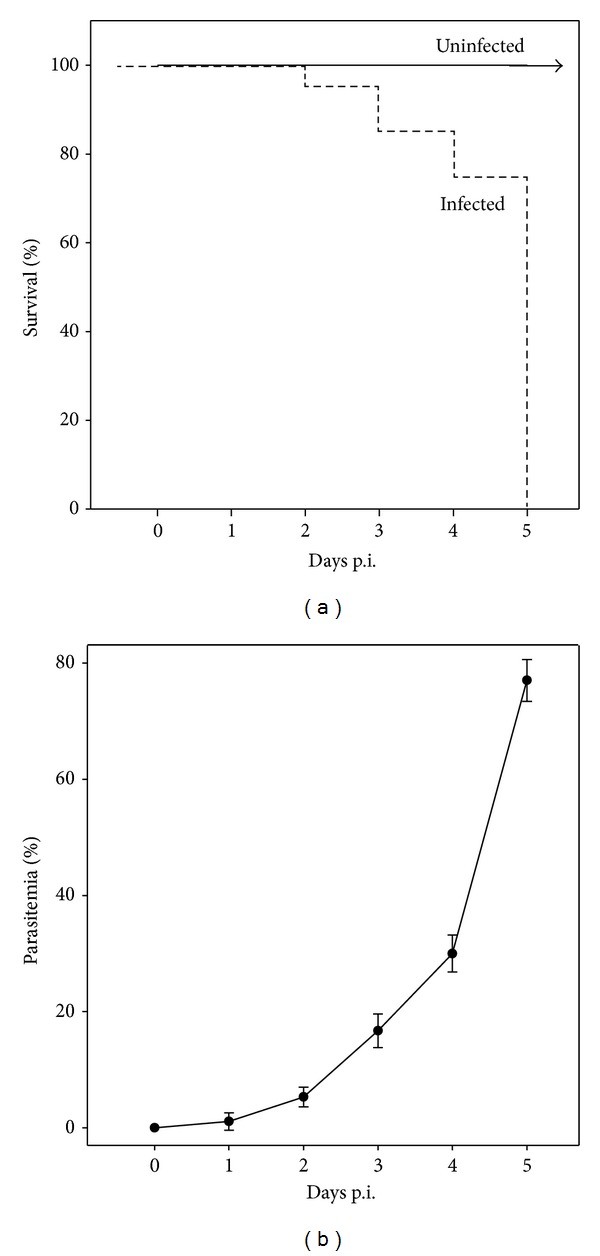
Survival (a) and parasitemia (b) of female gerbils (*n* = 12) infected with 5 × 10^6^ erythrocytes parasitized by* B. divergens*. All values are means ± SEM.

Challenge of gerbils with 5 × 10^6^ *B. divergens* infected erythrocytes induced a lethal outcome of the infection. No gerbils survived after day 5 p.i. (Figure 1). In this study and in our previous work, the parasitemia reached about 77% [20]. Symptoms of babesiosis clearly appeared on day 5 p.i., with shivering, fever, and bloody urine.

**Figure 2:**
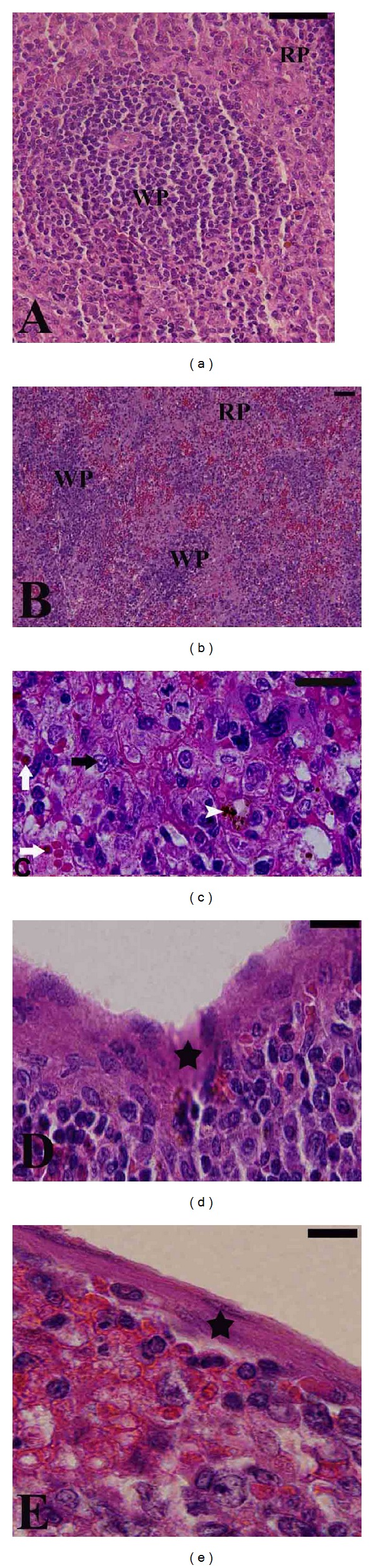
*Babesia divergens*-induced changes in spleen histology of gerbil. (a) Normal architecture of noninfected gerbil spleen. Red pulp (RP) and white pulp (WP) are separated; marginal zone and trabeculae are clearly observed. ((b) and (c)) Infected gerbil spleen on day 5 postinfection. WP is starting to fuse together; some spleen cells are vacuolated (black arrow), hemosiderin pigments are present (white arrow heads), and the splenic sinusoids contain infected erythrocytes (white arrow). (d) Normal spleen capsule (star). (e) Thin capsule from infected spleen of gerbil (star). Scale bar = 25 *μ*m.

**Figure 3:**
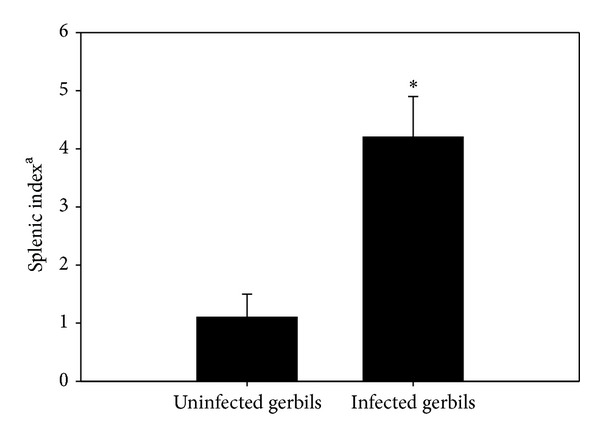
Increase in splenic index on day 5 postinfection with* Babesia divergens*. ^**a**^Ratio of spleen weight in mg/gerbil to body weight in g/gerbil.).

**Figure 4:**
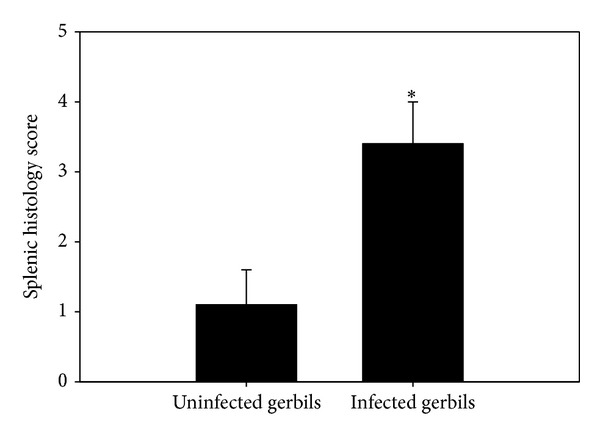
Histological score of the spleen. All values are means ± SD. *Significant data with respect to uninfected gerbils (*P* < 0.01).

Spleens of infected gerbils became enlarged and darker in color. Noninfected spleen was composed of white and red pulps surrounded by a capsule of dense connective tissue (Figure 2(a)). The white pulp was composed of a central, T-cell rich zone and a periarterial lymphoid sheath surrounded by B-cell-rich primary follicles. The white pulp was separated from the red pulp by the marginal sinus embedded in a layer of marginal zone lymphocytes. At maximum parasitemia on day 5 p.i., the white pulp enlarged due to cellular proliferation. The limit between white and red pulp started to disappear (Figures 2(b) and 2(c)), and the spleen increased in size. Vacuolation of some splenic cells was detected. Most of the cells were darkly stained and the sinusoidal spaces were large. This disorganization was due to hyperplasia of the lymphoid tissue. The capsule of the infected spleen became thinner (Figure 2(e)) when compared to the noninfected spleen capsule (Figure 2(d)). Spleen size increased as evidenced by the splenic index (Figure 3). In general,* B. divergens* induced a significant increase in the histological score of the splenic tissue as shown in Figure 4.

*B. divergens* infections also induced a highly significant increase in splenic MDA and nitrite/nitrate by approximately 3- and 2-fold, respectively (Table 1).

We determined some major components involved in the regulation of substances formed during oxidative stress such as GSH and catalase and superoxide dismutase, respectively (Table 2). Conspicuously, these substances were significantly downregulated by* B. divergens* infections.

Spleen damage was also recorded through the measurement of LDH and protein carbonyl content in spleen cells. These two markers of spleen injury were significantly increased (*P* < 0.001) due to* B. divergens* infection (Table 3).

Table 4 shows the effect of infection with* B. divergens *infected erythrocytes on gerbil cell cycle. The percentage of spleen cells at G_0_/G_1_ was significantly reduced while; S and G_2_/M phases were significantly elevated by the infection.

## 4. Discussion

The Mongolian gerbil (*Meriones unguiculatus*) is the main, fully susceptible experimental model used to study* B. divergens* infections [21], but unlike the bovine natural host, the gerbil develops acute and often fatal babesiosis.

*Babesia* parasites, like* Theileria* and malaria parasites, invade erythrocytes of infected animals, resulting in the destruction of parasitized erythrocytes [22]. During the early response to an initial infection, mononuclear phagocytes in the spleen would be expected to encounter merozoites or infected erythrocytes triggering an innate immune response as well as promoting a specific adaptive response. The role of the spleen in controlling infections is probably due to its phagocytic activity [23].

Histologic examination of spleen tissue of gerbils infected with* B. divergens* infected erythrocytes showed disturbed T-cell areas and changes in splenic architecture. The spleen is thought to form not only T- and B-cell dependent immune mechanisms, but also that site where parasitized red blood cells (pRBC) are destroyed by the same mechanisms which the spleen uses to remove senescent and other aberrant erythrocytes from circulation. Basically, pRBC are eliminated by phagocytes in the red pulp areas of the spleen, specifically in extravascular beds. These form an open circulation through which blood is percolated on the way from arterioles to venules [24]. The splenomegaly detected in infected gerbils was associated with expansion of both white and red pulp due to increased spleen size. This reaction is due to increased hematopoietic support [25] and increased numbers of macrophages [26]; the macrophages increase due to erythrophagocytosis [3].

The quantity of the destroyed erythrocytes is usually much higher than the degree of parasitaemia, suggesting that nonparasitized erythrocytes may also be damaged [27]. In experimental* B. rossi *infection, there was a marked decrease in haematocrit long before parasites were detectible in peripheral blood. This early change was hypothesized to be caused by hemodilution, splenomegaly, and sequestration in the spleen [28]. This mechanism may include autoimmune haemolysis [29], reduced red cell deformability [30], and increased oxidative damage [27, 31]. Also, numerous studies have demonstrated that a variety of inflammatory cells are induced or activated by various oxidant-generating enzymes to kill intracellular and extracellular parasites [26]. The reactive species are produced primarily to attack invading microorganisms by nitration, oxidation, and chlorination reactions. However, excess amounts of ROS can cause an injury to host cells and lead to tissue damage [30]. Highly reactive oxygen free radicals have a role in the pathogenesis of various parasitic infections including* Babesia*,* Leishmania*,* Hepatozoon*,* Ehrlichia*,* Theileria, *and* Plasmodium *parasites [27, 31, 32]. However, to the best of our knowledge, levels of spleen MDA have not been previously reported in gerbils infected with* B. divergens*. MDA is excreted in urine, blood, and other body fluids and therefore serves as a marker of lipid peroxidation and the presence of oxidative stress [33]. The results of the current study demonstrate that there is a significant increase in concentrations of MDA (*P* < 0.01) in gerbils with diagnosed babesiosis versus the healthy control group. Increased levels of MDA have been reported in* B. gibsoni *infection [10, 27] and in a mixed infection of* Ehrlichia canis *and* B. gibsoni *[32].

*Babesia *infection induced oxidative stress in spleen as evidenced by the decreased GSH, CAT concentration as well as increased formation of lipid peroxidation, and nitrite/nitrate. GSH is an excellent and potent endogenous antioxidant, which by scavenging various types of reactive radicals protects the cell from oxidative insults [34]. On encounter with reactive radicals, GSH stores may be depleted, leaving the cells with “compromised antioxidant defense system” against oxidant-induced injury.* B. divergens *infection depleted cellular GSH content. Decrease in cellular GSH content thus indicates generation of large quantity of ROS [6]. This fact is complicated due to the oxidation of protein as a result of ROS which leads to inhibition in the glutathione cycle system enzymes, glutathione redox pool [35].

Also, lactate dehydrogenase is a cytoplasmic enzyme present in essentially all major organ systems. The extracellular appearance of LDH is used to detect cell damage or cell death [36]. Moreover, infection with* B. divergens* significantly increased the formation of protein carbonyl; a measure of oxidative damage also correlated well with the percentage of parasitemia [6, 18].

Infection with some pathogens could alter the cell cycle [36]. Our study also demonstrated that the percentage of spleen cells in G_0_, G_1_, G_2_, and S phases was significantly altered due to infection.

*In Conclusion. B. divergens* infected erythrocytes could alter the spleen histopathology, cause cell cycle alteration, and induce oxidative stress in splenic tissue. Understanding of the pathogenesis induced in the spleen due to infection with* Babesia* is useful for both the management and the prevention of the infection.

## Figures and Tables

**Table 1:** Changes of malondialdehyde and nitrite/nitrate in spleen of gerbils infected with *B. divergens* (*n* = 8).

	Malondialdehyde (nmol/g)	Nitrite/nitrate (*μ*mol/g tissue)
Noninfected	6.7 ± 0.81	6.40 ± 1.01
Infected	19.9 ± 3.17∗	11.7 ± 2.60∗

Values are means ± SD. ∗Significant change at *P* ≤ 0.05 with respect to the noninfected control group.

**Table 2:** Changes of glutathione, catalase, and superoxide dismutase in splenic tissue of gerbils infected with *B. divergens* (*n* = 8).

	Glutathione (mmol/g spleen)	Catalase (U/g spleen)	Superoxide dismutase (U/g spleen)
Noninfected	7.4 ± 0.83	0.52 ± 0.01	14.2 ± 0.42
Infected	4.1 ± 0.26∗	0.25 ± 0.01∗	9.3 ± 0.7∗

Values are means ± SD. ∗Significant change at *P* ≤ 0.05 with respect to the noninfected control group.

**Table 3:** Changes of lactic acid dehydrogenase activity and protein carbonyl content in splenic tissue of gerbils infected with *B. divergens* (*n* = 8).

	Lactic acid dehydrogenase activity (U/g protein)	Protein carbonyl content (nmol/g protein)
Noninfected	168.99 ± 6.76	3.83 ± 0.12
Infected	312.91 ± 5.92∗	17.40 ± 1.95∗

Values are means ± SD. ∗Significant change at *P* ≤ 0.05 with respect to the noninfected control group.

**Table 4:** Cell cycle of spleen cells of gerbils infected with *B. divergens* infected erythrocytes.

Phase	Noninfected gerbils	Infected gerbils
G_0_/G_1_	97.14 ± 0.33	77.89 ± 0.42∗
S phase	2.5 ± 0.011	14.60 ± 0.032∗
G_2_/M	0.36 ± 0.012	7.51 ± 0.023∗

Values are means ± SEM. ∗Significant change at *P* ≤ 0.05 with respect to control group.
